# The Quality of Surgical Care at a Newly-Started Healthcare Center and Its Objective Assessment by the Modified Jabalpur-Portsmouth Physiological and Operative Severity Score (J-POSSUM) in Northern India

**DOI:** 10.7759/cureus.93322

**Published:** 2025-09-27

**Authors:** Niraj Srivastava, Sunita Singh, Rounak Mehrotra, Mukesh Shukla, Somteertha Ray, Amit Gupta

**Affiliations:** 1 General Surgery, All India Institute of Medical Sciences (AIIMS), Raebareli, IND; 2 Pediatric surgery, All India Institute of Medical Sciences (AIIMS), Raebareli, IND; 3 Community and Family Medicine, All India Institute of Medical Sciences (AIIMS), Raebareli, IND

**Keywords:** j-possum, o/e ratio, possum, p-possum (the portsmouth physiological and operative severity score for the enumeration of mortality and morbidity), quality improvement, surgical audit

## Abstract

Background

Risk-adjusted mortality and morbidity provide more accurate and insightful data on the quality of care any healthcare system delivers. The Physiologic and Operative Severity Score for the Study of Mortality and Morbidity (POSSUM) and Portsmouth-POSSUM (P-POSSUM) calculate the risk for operative morbidity and mortality, which can help patients and their families make informed decisions about surgery, complications, and quality of care audits. The study aimed to assess existing surgical services objectively via the validated POSSUM equation in a developing tertiary health care institute.

Methods

The study was a hospital-based ambispective study at a newly started hospital in Northern India from February 2022 to February 2024 among patients aged 12-75 years. The study was conducted in two phases. The first phase involved a retrospective audit of patient records, validation/Correction of the POSSUM equation, and measures to improve the quality of patient care. The second phase determined the prediction of morbidity via the modified POSSUM equation and outcome assessment after implementation of patients' quality of care corrective measures.

Morbidity was considered if any intraoperative and/or postoperative complications occurred within 30 days of the index operation. Exclusion criteria were severely immunocompromised patients, polytrauma patients, and patients with transfusion-related reactions. The surgical procedures were categorised according to the surgeon's convenience into four categories to compare the difficulties of surgical procedures. If a patient developed multiple complications, they were considered as one patient.

Results

The POSSUM and P-POSSUM equations for morbidity and mortality (n=217) in phase I showed the observed/expected ratio 3.42 (p=0.04) for patients categorised in the minor surgeries. So, Jabalpur-POSSUM (J-Possum) was recalculated by a correction factor of 0.619 for morbidity (predicted risk is <40%) and 0.257 (predicted risk is <10%). The J-POSSUM was plotted against morbidities to reassure predictive accuracy (p=0.00).

Mean physiological and operative scores of phase II (n=470) patients were comparable with those of phase I (n=217) (p=0.11 and p=0.52). The number of patients having predicted risk of increased morbidity increased significantly in phase II (p=0.00), but complication rates decreased significantly from 69/214 (32.2%) to 71/470 (15.10%) (p=0.02). The predicted J-POSSUM equation suggested a significant decrease in intraoperative/ postoperative morbidities in phase II (p=0.00 and p=0.016), despite the increased number of patients having an increased predicted risk of morbidity in phase II (p=0.00).

The expected increase in the number of patients at risk for mortality was enhanced on J-POSSUM (p=0.016). In phase I and II, mortality was 2/214 (0.09%) and 7/470 (1.5%), respectively. The P-POSSUM equation for mortality cannot be validated in the study population because of undersampling.

Conclusion

The author suggests the use of J-POSSUM as a validated benchmarking score in Indian settings to predict morbidities, especially in elective minor, intermediate, and major cases. Due to undersampling, the P-POSSUM equation for mortality could not be used in the current study.

## Introduction

The surgical audit plays a crucial role in identifying predictions and limits, which depend on the number of operations performed and the number of adverse outcomes. Unlike crude mortality, risk-adjusted mortality and morbidity provide more accurate and insightful data on the quality of care any healthcare system delivers [1]. It's crucial to avoid overreacting to adverse outcomes and focus on carefully examining the causes of quality deficiencies [2]. The result of surgical intervention, be it death, complications, uncomplicated survival, or long-term morbidity, is not solely dependent on the surgeon's abilities in isolation. The patient's physiological status, the disease that requires surgical correction, the nature of the operation, and the preoperative and postoperative support services play a significant role in determining the outcome [3, 4]. It is evident to surgeons worldwide that raw mortality and morbidity rates do little to expound these differences and that such statistics are, at best, inaccurate and dangerous.

When taken to an extreme, mortality rates can achieve what appears to be a self-fulfilling prophecy. The unit that selects only low-risk cases achieves a low mortality rate and attracts more patients. In contrast, the unit that can choose not only low-risk cases but is left with a worsening case mix, and their performance as judged by mortality rate will appear to deteriorate further over time [5].

A multivariate discriminant analysis was adapted by surgeons for the surgery-related complications risk assessment to develop a comprehensive scoring system that accurately predicts 30-day mortality and morbidity rates. The Physiological and Operative Severity Score for the Enumeration of Mortality and Morbidity (POSSUM) audit system was initially designed to facilitate easy, rapid, and wide application across the general surgical spectrum. A POSSUM-based audit system is applicable in most healthcare systems in both elective and emergency settings. The POSSUM calculates the risk for operative morbidity and mortality, which can help patients and their families make informed decisions about surgery and reassure them about its benefits [6]. The POSSUM assesses morbidity and mortality for patients older than 12 years [6]. Various authors have already validated this score [6]. Healthcare workers regularly want to improve existing services in a developing tertiary institute. The best way to do this is to do an audit. Hospitals worldwide have adopted the same procedure to deliver better-quality care to patients. There might be a caveat from patients, clinicians, nursing, infrastructure, or scarcity of resources. The study aimed to assess existing surgical services objectively via the validated POSSUM equation in a developing tertiary healthcare institute.

## Materials and methods

The study was an ambispective study at a newly started healthcare centre in Northern India from February 2022 to February 2024.

Inclusion and exclusion criteria

Patients aged 12 to 75 years who underwent either emergency or elective surgeries and completed 30 days of follow-up were included in the study. We excluded severely immunocompromised patients, viz, patients on cancer chemotherapy or suffering from human immunodeficiency syndrome, polytrauma patients, patients operated in a daycare or minor operation theatre, and patients who lost 30 days of follow-up. Other complications excluded were transfusion-related reactions, instrument failure, bad scars, or hypertrophic scars. 

Morbidity

The morbidity was considered if any intraoperative and postoperative complications (occurring within 30 days of the index operation) were counted. Intraoperative complications observed were significant hemorrhage (loss of blood volume more than acceptable for weight, considering a healthy, average, and poor body-built patient can bear 30%, 20%, and 10% loss of blood volume, respectively) [7], additional requirement inotropes to maintained patient's hemodynamics during surgery, bradycardia needs drug or cardiopulmonary resuscitation, damage to vital structures adjacent to diseased organ, and difficulty in extubation. Operative complications were surgical-site infection (Center for Diseases Classification definition-2018) [8], wound dehiscence, dyselectrolytemia (raised or decreased serum sodium, potassium, calcium or magnesium), wound dehiscence (partial or total wound dehiscence), fever (temperature > 38.3 C) with known source related to surgery or during index hospital admission; septicemia, systematic inflammatory response syndrome (SIRS), septic shock, multiple organ failure syndrome (MODS) (the American College of Chest Physician/Society of Critical Care Medicine-sponsored definitions) [9]; catheter-associated urinary tract infection (CDC-2018 definition) [10]; central line-related bloodstream infection (CDC-2018 definition) [11]; ventilator-associated pneumonia (CDC-2018 definition) [12]; and delayed wound healing and readmission within a month due to surgery-related complications. The overlapping (concordant) and nonoverlapping (nonconcordant) complications were counted as one for an individual.

The POSSUM equation for morbidity and mortality was calculated using physiological scores (PS) and operative scores (OS). PS and OS were estimated using patients' physiological parameters at the time of surgery and intraoperative findings. Data for technical difficulties, intra-procedural complications, and postoperative complications were collected.

The physiological part of the score included 12 variables, each divided into four grades with an exponentially increasing score (1, 2, 4, and 8). The physiological variables were those apparent at the time of surgery and included clinical symptoms and signs, biochemical and hematological investigations, and electrocardiographic changes (Table 1).

**Table 1:** Preoperative P-POSSUM equation calculation based on physiological and operative scores COPD - chronic obstructive pulmonary disease; OS - operative score; PS - physiological score; BP - blood pressure; WBC - white blood cell count; JPV - jugular venous pressure; P-POSSUM - Portsmouth-Physiologic and Operative Severity Score for the Study of Mortality and Morbidity

Preoperative patient physiological score					
Score		1	2	4	8
Cardiac Signs, chest X-rays		Normal	Cardiac drugs or steroids	Pedal edema; patients on warfarin therapy, borderline cardiomegaly	JVP cardiomegaly
Respiratory signs, hest X-rays		No dyspnea, normal	Exertional dyspnea, mild COPD	Limiting Dyspnea or climbing stairs, moderate COPD	Dyspnea at rest, fibrosis consolidation on X-rays
Systolic BP at the time of admission, mm Hg		110-130	131-170 100-109	≥ 171 90-99	≤89
Pulse (beats/min)		50-80	81-100 40-49	101-120	≥ 121 ≤39
Coma score		15	12-14	9-11	≤8
Urea nitrogen, mmol/L		<7.5	7.6-10	10.1-15	≥15.1
Na, mEq/Lit		>136	131-135	126-130	≤125
K, mEql/Lit		3.5-5	3.2-3.4 5.1-5.3	2.9-3.1 5.4-5.9	≤2.8 ≥6
Hb, g/dL		13-16	11.5-12.9 16.1-17	10-11.4 17.1-18	≤9.9 ≥18.1
WBC (x10^12^/Lit)		4-10	10.1-20 3.1-3.9	≥20.1 ≤3	….
Electrocardiogram		Normal	…	AF (60-90)	Five ectopic beats/min, Q waves or ST/T wave changes Any other abnormal rhythm
Intraoperative patient operative score (OS)					
Score	1		2	4	8
Operative magnitude	Minor		Intermediate	Major	Major+
No. of operations within 30d	1			2	>2
Blood loss per operation, in ml	<100		101-500	501-999	00
Contamination	No		Incised wound, i.e., stab	Minor contamination or necrotic tissue	Gross contamination or necrotic tissue
Presence of malignancy	No		Primary cancer only	Node metastases	Distant metastases
Timing of operation	Ee			Emergency resuscitation possible <48h	Emergency immediate, <6h

A score of one was allocated if a particular variable was unavailable. Some variables were assessed using clinical symptoms, signs, or changes in chest radiographic findings. The minimum score was 12, with a maximum score of 88. POSSUM scoring was done via an online calculator for POSSUMScoring. The following equation was used to predict the risk of morbidity and mortality in individual patients.

POSSUM equation for morbidity was Log (R/1-R) = −5.91 + (0.16×physiological score) + (0.19×operative score); where R is the predicted risk of morbidity.

POSSUM equation for mortality was Log (R/1-R) = −7.04 + (0.13×physiological score) + (0.16×operative score); where R is the predicted risk of mortality. Large values suggest poor fit, with calibration considered poor if p ≤0.05.

P-P POSSUM equation for mortality: Log (R2/1-R2) = −9.065+ 0.1692xPS +0.15550x operative score (OS), where -0.965 is the correction factor for P-POSSUM.

The study was conducted in two phases. The first phase was a retrospective audit of operative records and morbidity to assess the magnitude of the Complications, Validation/Correction of the POSSUM equation, and continuous measures to improve the existing surgical care-related services to improve the quality of care.

The second phase was the prospective collection of patient data, to determine the prediction of morbidity via the modified POSSUM equation and surgical outcome assessment after implementation of patients' quality of care corrective measures. 

The predictive accuracy of morbidity and mortality of the POSSUM equation in the study population was checked by calculating PS and OS from the operative records of patients operated on from February 2022 to September 2022. Actual postoperative morbidity and mortality were also recorded. The receiver operative curve (ROC) for the prediction accuracy of POSSUM was drawn to ensure the accuracy of the POSSUM equation in our study population. The surgical procedures were categorized according to the surgeon's convenience into various categories to compare the difficulties in surgical procedures (Table 2).

**Table 2:** Patients categorized based on surgical risk stratification for morbidity and mortality MRM - modified radical mastectomy; IPOM - intraperitoneal onlay-mesh; TAPP - transabdominal preperitoneal; MIPH - minimal invasive surgery for prolapsed hemorrhoids

Minor	Intermediate	Major	Major+
Standard orchidopexy/ orchiectomy/ hydrocelectomy, internal sphincterotomy for anal fissure, hemorrhoidectomy (MIPH and Milligan-Morgan), granulomatous mastitis, repair of epigastric hernia/ inguinal hernia/ umbilical hernia/ paraumbilical hernia, excision of giant fibroadenoma/ giant lipoma, Hadfield operation for ductal ectasia, subcutaneous mastectomy for gynecomastia, metoplasty for meatal stenosis, high inguinal orchidectomy	Hypospadias surgery, laparoscopic cholecystectomy, excision of urachus/umbilical mass/ brachial sinus, laparoscopic appendectomy IPOM for incisional hernia, lobectomy for thyroid swellings, excision of arteriovenous malformation/ lymphangioma, excision of giant phyllodes tumour, flap rotation for pilonidal sinus, fistulectomy for low fistula-in-ano	Superficial parotidectomy for pleomorphic adenoma, wide local excision of squamous cell carcinoma, burst liver abscess, acute sigmoid/intestinal volvulus, TAAP for recurrent inguinal hernia, recurrent or high fistula in ano multiple soft tissue abscess/ extensive cellulitis/extensive necrotising fasciitis/ diabetic foot with septicemia, cystojejunostomy for pseudo-pancreatic cyst, elective laparotomy for benign diseases laparoscopic suture/mesh rectopexy for rectal prolapse/ cystocele repair, laparoscopic cardiomyotomy with fundoplication, ileostomy/colostomy closure, congenital/acquired diaphragmatic hernia repair, MRM for breast carcinoma, total/ subtotal thyroidectomy	Intestinal obstruction, emergency laparotomy for perforation peritonitis, choledochal cyst excision with hepaticojejunostomy, abdominoperineal resection for colorectal carcinoma, R-2 gastrectomy for carcinoma of the stomach, radical cholecystectomy for carcinoma of the gall bladder, excision of retroperitoneal sarcoma

The interventions to improve quality of care were the identification of factors causing under-performance in the delivery of appropriate surgical care via continuous feedback from nurses, patients, and peer colleagues. The team of authors made constant efforts to fill the gaps in delivering surgical care to the patients, viz introducing (a) surgical care bundles [13, 14]; (b) WHO time-out practices [15]; (c) patient education materials, (d) collaboration of different ranks of healthcare workers with multidisciplinary collaboration, (e) modifying attitudes and approaches of nursing staff to address patient safety issues, (f) provision of a blame-free environment [16, 17]; (g) performing educational activities to enhance psychomotor skills; and (h) implementing a simulator-based sensitisation module to residents and nursing staff [18].

The first and second phases contained a continuous peer review and feedback mechanism to readdress the health care delivery-related problems.

Data collection

In the first phase, data was collected from patients' medical records, and the POSSUM equation was used for PS and OS calculation.

Outcome measures

The crude and risk-adjusted morbidity and mortality rates were compared between the two phases. Intraoperative and postoperative complications together are calculated to consider morbidity.

Statistical analysis

Data analysis was performed using IBM SPSS Statistics version 26 (IBM Inc., Armonk, NY, USA). Descriptive statistics, such as frequency and percentage for categorical variables, were determined. Chi-squared was used to compare the baseline variables in the two phases. The predicted risk of morbidity (intra-operative/postoperative complications) was calculated using a standard equation based on exponential analysis. The predicted risk of morbidity, i.e., the risk of any intra-operative or postoperative complication, was calculated using the POSSUM equation on morbidity. The predicted/estimated risk was plotted against the observed intraoperative/postoperative complications for plotting the receiver operating curve (ROC).

## Results

Phase one

Three hundred patients underwent emergency/elective surgeries in phase one. After data cleaning and removal of duplications, 214 patients' records were used for PS and OS calculation. The POSSUM and P-POSSUM equations for morbidity and mortality were calculated. In phase one, the O/E ratio for morbidities was 3.42, which did not significantly predict outcome (p=0.04), especially for patients categorised as undergoing minor surgeries. Therefore, the correction factor for Jabalpur POSSUM (J-POSSUM) was applied. These factors were 0.619 for morbidity if the predicted risk is <40% and 0.257 if the predicted mortality risk is <10% [18, 19] (Table 3).

**Table 3:** Comparison of POSSUM predicted morbidity and mortality with the observed morbidity and mortality in the two phases A p-value of <0.05 is considered statistically significant. COPD - chronic obstructive pulmonary disease; POSSUM - Physiologic and Operative Severity Score for the Study of Mortality and Morbidity

Risk group (n)	Mean risk scores	Expected	Observed	No. of patients	O:E	p-value
Phase one POSSUM morbidity (N=214)						
Minor	7.90	7	24	80	3.42	0.04
Intermediate	9.80	12	17	118	1.41	0.08
Major	32.93	4	5	13	1.25	0.17
Major +	21.9	1	1	3	NA	NA
Phase two POSSUM morbidity (N=470)						
Minor	7.98	10	3	116	0.3	0.20
Intermediate	5.90	16	3	271	0.18	0.12
Major	24.4	19	8	75	0.42	0.68
Major +	31.5	3	1	8	0.33	0.56
Phase one POSSUM mortality (n=214)						
Minor	0.55	1	0	80	0	0.31
Intermediate	0.29	1	0	118	0	0.31
Major	5.001	1	0	13	0	0.31
Major +	0.35	0	1	3	NA	NA
Phase one P-POSSUM mortality (N=214)						
Minor	0.036	0	0	80	0	2.00
Intermediate	0.023	1	0	118	0	0.31
Major	0.54	1	0	13	0	0.31
Major +	0.02	0	1	3	NA	NA
Phase two POSSUM mortality (N=470)						
Minor	2.06	3	1	116	0.33	0.56
Intermediate	2.2	6	1	271	0.16	0.18
Major	9.9	8	1	75	0.125	0.03
Major +	12.3	1	0	8	0	0.31
Phase two P-POSSUM mortality (N=470)						
Minor	0.59	1	1	116	1	0.31
Intermediate	1.02	3	1	271	0.33	0.56
Major	6.62	5	1	75	0.2	0.17
Major +	10.7	1	0	8	0	0.31

Efficacy of the J-POSSUM Score in Predicting the Risk of Morbidity and Mortality ROC Curves

**Figure 1:**
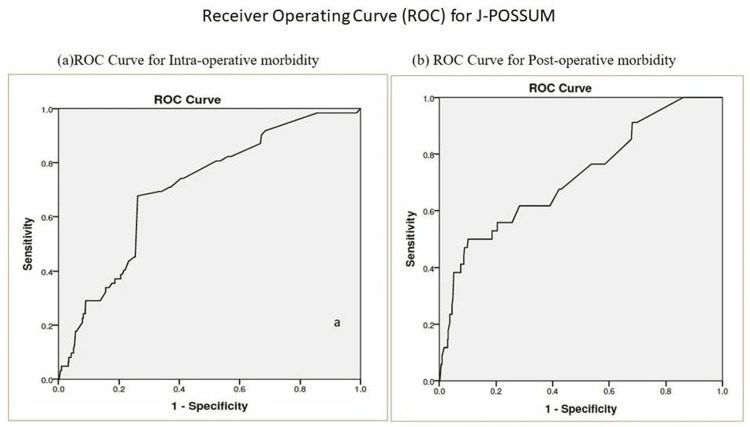
The receive operating curve for morbidity predicted by the J-POSSUM equation The receiver operating curve shows intraoperative morbidity predicted by the J-POSSUM equation. (a) The area under the curve for intraoperative complications was 0.705, and for (b) postoperative complications, it was 0.719 (p-value 0.00), showing the J-POSSUM score's good prediction ability for morbidity. A p-value of <0.05 was considered statistically significant J-POSSUM - Jabalpur-Physiologic and Operative Severity Score for the Study of Mortality and Morbidity; ROC - receive operating curve

The J-POSSUM equation was recalculated for individual risk of morbidity and mortality and plotted (ROC curve) against the morbidity for intraoperative and postoperative complications. The area under the curve for intraoperative and postoperative complications was 0.705 and 0.719, respectively, with a significant p-value (0.00) (Figure 1).

ROC curves in Figure 1 showed, the J-POSSUM equation for morbidity could be considered a good predictor for intraoperative/ postoperative complications in the study population.

Phase two

Five hundred fifty patients who underwent emergency/elective major surgeries in phase two were prospectively followed with the application of J-POSSUM for risk prediction of morbidities. After data cleaning and removal of duplicates, 470 patients were included for data analysis. Table 3 compares the demography and surgical profile of the two phases: in the first phase, the mean age of patients was 40.24 ± 18.6 years, and the male-to-female ratio was 6.07:1. In the second phase, the mean age of patients was 43.24 ± 11.6 years, and the male-to-female ratio was 4.18. Most (33/684, 77.9%) of the procedures were elective surgeries, while 151/684 (22.07%) were emergency surgeries. The profiles of patients who underwent surgery in two phases were slightly different. However, the mean PS±SD and OS±SD in two phases were not different (PS (p=0.11) and OS (p=0.52)). 

**Table 4:** Demography, comorbidities, physiological scores, operative scores and clinical profile of patients A p-value of <0.05 was considered statistically significant; unpaired t-test. COPD - chronic obstructive pulmonary disease

Patient characteristics	Phase one, n=214 (%)	Phase two, n=470(%)	p-value
Age-group			
12-20	10 (4.7)	71 (15.1)	0.00
21-30	44 (20.6)	88 (18.7)	
31-40	58 (27.1)	81 (17.2)	
41-50	44 (20.6)	107 (22.8)	
51-60	34 (15.9)	60 (12.8)	
>60	24 (11.2)	63 (13.4)	
Gender			
Male	182 (85.0)	377 (80.2)	0.078
Female	32 (15.0)	93 (19.8)	
Co-morbidity status			
Diabetes	31 (14.5)	36 (7.7)	0.008
Congestive heart failure	5 (2.3)	1 (0.2)	0.013
Hypertension	30 (14.0)	41 (8.7)	0.042
History of transient ischemic attack	2 (0.9)	0 (0.0)	0.098
Hypothyroidism	11 (5.1)	6 (1.3)	0.006
Peripheral vascular diseases	0 (0.0)	1 (0.2)	0.50
H/o Cerebrovascular accident or neurological-deficit	0 (0.0)	1 (0.2)	0.50
COPD	3 (1.4)	1 (0.2)	0.02
Frailty status			
1-2	210 (98.1)	408 (86.8)	0.00
0	4 (1.9)	62 (13.2)	
Physiological score			
11-15	155 (72.4)	350 (74.5)	0.11
16-20	24 (11.2)	75 (16.0)	
21-25	21 (9.8)	16 (3.4)	
>26	14 (6.5)	29 (6.2)	
Operative score			
6-10	141 (65.9)	323 (68.7)	0.52
11-15	66 (30.8)	118 (25.1)	
>16	7 (3.3)	29 (6.1)	

Morbidity

The morbidity of patients in two phases was calculated by intraoperative and postoperative complications. No intraoperative bradycardia, arrhythmias, anaphylaxis, or damage to named vessels occurred among patients in either phase. The first phase crude morbidity (total intraoperative and postoperative complications/ total surgeries) rate was 88/214 (41.1%), and the second phase crude morbidity rate was 130/470 (27.6%).

**Table 5:** Comparison of patient number falling in predicted risk of morbidity and mortality based on the J-POSSUM/P-POSSUM equation on morbidity and mortality and P-POSSUM equation for mortality in phase one and phase two Comparison of patient number falling in predicted risk of morbidity and mortality based on J-POSSUM/P-POSSUM equation for morbidity and mortality, and the P-POSSUM equation for mortality in the first and second phases. POSSUM - Physiologic and Operative Severity Score for the Study of Mortality and Morbidity; J-POSSUM - Jabalpur-POSSUM; P-POSSUM - Portsmouth-POSSUM

Predictive risk	Phase one (n=214)	Phase two (n=470)	Chi-squared	p-value
	Risk of morbidity (J-POSSUM)			
0.00-0.25 (very-low risk) (n=600)	178 (83.2)	422 (89.8)	25.35	0.00
0.26-0.50 (low risk) (n=36)	24 (11.2)	12 (2.6)		
0.51-0.75 (moderate risk) (n=11)	5 (2.3)	6 (1.3)		
0.76-1.00 (High risk) (n=37)	7 (3.3)	30 (6.4)		
	Risk of mortality (J-POSSUM)			
0.00-0.25 (very-low risk) (n=600)	214 (100.0)	448 (95.3)	10.35	0.016
0.26-0.50 (low risk) (n=36)	0 (0.0)	1 (0.2)		
0.51-0.75 (moderate risk) (n=11)	0 (0.0)	12 (2.6)		
0.76-1.00 (High risk) (n=37)	0 (0.0)	9 (1.9)		
	Risk of mortality (P-POSSUM)			
0.00-0.25 (very-low risk) (n=600)	214 (100.0)	461 (98.1)	4.15	0.24
0.26-0.50 (low risk) (n=36)	0 (0.0)	1 (0.2)		
0.51-0.75 (moderate risk) (n=11)	0 (0.0)	1 (0.2)		
0.76-1.00 (High risk) (n=37)	0 (0.0)	7 (1.5)		

If a patient developed multiple complications, they were considered complications as one patient. The number of patients having predicted risk of increased morbidity increased significantly in phase two (p=0.00), but complication rates decreased significantly from 69/214 (32.2%) to 71/470 (15.10%) (p=0.02). The predicted J-POSSUM equation suggested a significant decrease in intraoperative/ postoperative morbidities in phase two (p=0.00 and p=0.016) despite the increased number of patients having increased predicted risk of morbidity in phase two (p=0.00).

The crude mortality rate was total deaths/admissions x 100 (13%). The crude mortality rate in the first phase was 0.09% (2/214), and in the second phase. The crude mortality rate was 7/470 (1.5%). Patients who underwent emergency laparotomy were the most common. The table showed a significant increase in the number of patients who have a predicted risk of increased morbidity in phase two (p=0.00). The calculations for the expected increase in the number of patients for mortality risk do not improve significantly in phase two on P-POSSUM (p=0.24), but are enhanced on J-POSSUM (p=0.016)

Table 6 suggests the association between the observed complications in two phases (intra-operative/post-operative) and the predicted risk (expected complication). The predictive risk of morbidity for intraoperative and postoperative complications was calculated using the J-POSSUM equation (p=0.00).

**Table 6:** Comparison of intra-operative complications in phase one and phase two (N=684) A p-value of <0.05 was considered statistically significant.

Complications	Phase one, n=214 (%)	Phase two, n=470(%)	p-value
Intra-operative complications			
Significant intraoperative hemorrhage	5 (2.3)	2 (0.42)	0.32
Intraoperative requirement of inotropes to stabilize vitals	6 (2.8)	1 (0.2)	0.00
Bradycardia	11 (5.1)	3(0.0)	0.00
Damage to adjacent vital structures	0 (0.0)	1 (0.2)	0.50
Difficulty in extubation	3 (0.9)	9 (1.9)	0.34
Post-operative complications			
Surgical-site infection	26 (12.1)	8 (1.7)	0.00
Dys-electrolytemia	6 (2.8)	8 (1.7)	0.34
Wound dehiscence	5 (2.3)	16 (3.4)	0.45
Septicaemia	0 (0.0)	11 (2.3)	0.02
Fever with a known source	1 (0.5)	4 (0.9)	0.58
Catheter-associated urinary tract infection (CAUTI)	2 (0.9)	0 (0.0)	0.03
Central line-related bloodstream infection (CLABSI)	0 (0.0)	4 (0.9)	0.83
Systematic inflammatory response syndrome (SIRS)	1 (0.5)	1 (0.2)	0.56
Multiple organ failure syndrome (MODS)	1 (0.5)	1 (0.2)	0.56
Septic shock	1 (0.5)	2 (0.4)	0.93
Readmission within a month due to index surgery-related complications	1 (0.5)	0 (0.0)	0.13
Total morbidity (Total complications/ Total patients underwent surgery)	69/214 (32.2)	71/470 (15.10)	0.02
Mortality	2 (0.5)	7 (0.2)	0.56

Crude complication rates showed a significant decrease in rate from 69/214 (32.2%) to 71/470 (15.10%) (p=0.02). The overall most common intraoperative complication was bradycardia (n=14), followed by intraoperative hemorrhage (n=7) and the need for ionotropic support (n=7). The patients developed intraoperative bradycardia, and the need for inotropes to stabilize the vitals significantly decreased in the second phase (p=0.00). The most common postoperative complication was surgical site infection (n=26), followed by dyselectrolytemia. In the 30 days of postoperative follow-up, there were no severe chest infections, unplanned re-do surgeries, deep vein thrombosis, pulmonary embolism, cerebrovascular accidents, myocardial infarction, renal failure, or adverse drug reactions in either phase. In phase two, there was a significant decrease in surgical site infection (p=0.00) and CAUTI frequency (p=0.03). The postoperative septicemia is significantly increased in the second phase (p=0.02), with an insignificant increase in dyselectrolytemia (p=0.34), wound dehiscence (p=0.45), fever with known source (p=0.58), and CLABSI (p=0.83).

The predicted POSSUM equation suggested a significant decrease in intraoperative/ postoperative morbidities (complications) in phase two (p=0.00 and p=0.016) despite having comparable mean OS and PS (p=0.11 and p=0.52).

In Table 7, the predicted risk of morbidity (stratified into four categories according to severity from very-low to high) is shown. The Chi-squared test was applied to study the association between these four predicted categories with the occurrence of actual observed complications (intra-operatively and post-operatively). A significant association was found between the independent variable (predictive score category) and the outcome of interest (complication). In 13.5% and 21.6% of patients in the high-risk category, intraoperatively and postoperatively, respectively, complications were actually observed. 600 patients were expected to have a very low risk (0.00-0.25) for any morbidity, and only 59 (42+17)/600 (9.83 %) of these patients had intraoperative or postoperative complications. A total of 36 patients were expected to have low risk (0.26-0.50) for any morbidity, and 10 (4+4)/30 (33.3%) of them had either an intraoperative or postoperative complication. A total of 11 patients were expected to have a moderate risk (0.51-0.75) for any morbidity, and 9 (4+5)/11 (81.8%) of them had either intraoperative or postoperative complications. A total of 37 patients were expected to have a high risk (0.76-1.00) for any morbidity, and 14/37 (37.8%) of them had either intraoperative or postoperative complications.

**Table 7:** Association between post-operative and intra-operative complications with predicted risk of morbidity (J-POSSUM) A p-value of <0.05 was considered statistically significant. J-POSSUM - Jabalpur-Physiologic and Operative Severity Score for the Study of Mortality and Morbidity

Predictive risk using J-POSSUM	Complications present n (%)	Complications absent n (%)	Chi-squared	p-value
Intra-operative complications				
0.00-0.25 (very-low risk) (n=600)	42 [7.0]	558 [93.0]	26.85	0.00
0.26-0.50 (low risk) (n=36)	6 [16.7]	30 [83.3]		
0.51-0.75 (moderate risk) (n=11)	4 [36.4]	7 [63.6]		
0.76-1.00 (high risk) (n=37)	5 [13.5]	32 [86.5]		
Post-operative complications				
0.00-0.25 (very-low risk) (n=600)	17 [2.8]	583 [97.2]	68.55	0.00
0.26-0.50 (low risk) (n=36)	4 [11.1]	32 [88.9]		
0.51-0.75 (moderate risk) (n=11)	5 [45.5]	6 [54.5]		
0.76-1.00 (high risk) (n=37)	8 [21.6]	29 [78.9]		

Mortality-acquired pneumonia and ventilator-associated pneumonia were the most common causes of mortality. The second most common cause of mortality was septicemia. The low mortality rate in the study did not provide definitive information. The P-POSSUM equation for mortality cannot be relied on because of undersampling in both phases.

## Discussion

Lower-middle-income (LMICs) and lower-income (LICs) countries sometimes suffer due to lower-quality healthcare, with nearly eight million people dying each year due to low-quality healthcare systems and up to 2.6% loss of potential GDP in LICs due to these deaths. [20] In 1990, the Institute of Medicine defined quality as "The degree to which Health Services for individuals and populations increase the likelihood of desired health outcomes and are consistent with current professional knowledge". Some studies defined postoperative adverse events as any deviation from the expected recovery from surgery [21]. The government's focus on strengthening health organisations requires addressing surgical capacity to understand how to measure quality for surgical facilities.

The Acute Physiology and Chronic Health Evaluation (APACHE) system and the Goldman Index of Cardiac Risk, Surgical APGAR Scores were statistically sound systems to improve surgical care [21, 22]. APACHE II is a surgical risk assessment tool that is applied to intensive care patients to assess mortality risk [3]. The POSSUM system of surgical audit was first used in the United Kingdom and Europe, and the Veterans' Administration National Surgical Quality Improvement Program [22, 23]. The POSSUM is more comprehensive than the Surgical Apgar Score (using intraoperative parameters only)[24]. The POSSUM uses preoperative parameters. The American College of Surgeons National Surgical Quality Improvement Program for adults (ACS NSQIP) and Pediatrics (ACS NSQIP) risk calculators are newer, similar assessment tools. Still, they have not yet been rigorously validated as POSSUM. Thus, POSSUM Scoring is validated and utilized to predict mortality and morbidity in various surgeries [2-13]. POSSUM has been tested as an accurate study tool for predicting morbidity and mortality in emergency and elective surgeries in Western countries [4]. There are studies on procedure-specific POSSUM models for colorectal (CR-POSSUM), vascular (Vascular-POSSUM), and esophagogastric surgeries (O-POSSUM, O for oesophagogastric). POSSUM is an accurate predictor of postoperative morbidity for surgeries in inflammatory bowel disease patients, with over-prediction for mortality [25]. Further surgery for hip fractures and rectal cancers also showed the reliability of the O-POSSUM scale in predicting mortality [9, 26]. The POSSUM score can independently predict long-term survival [27].

 The limitations of POSSUM have been questioned in patients with advanced stages of cirrhosis, so we excluded patients with decompensated liver cirrhosis [19, 28]. A few authors from India calculated a correction factor for low-risk patients to predict mortality and morbidity [19]. In our first phase study population, the original POSSUM-assisted O/E ratio underestimated the prediction of morbidities for minor surgeries. Consequently, J-POSSUM with correction factors was used in our study population, and the ROC curves were plotted to confirm predictive risk in the study population.

The postoperative rate of septicemia, dyselectrolytemia, wound dehiscence, fever with known source, and CLABSI frequency increased in the second phase in our study population. One possible reason might be that high-risk cases were also admitted and treated with the expansion of intensive care services. 

The developed countries have been developing scoring systems to compare and collaborate on data within their institutes, e.g., ACS NSQIP, ACS NSQIP-P, Agency for Healthcare Research and Quality Patient Safety Indicators (AHRQ-PSI) [28, 29, 30]. These scores also help to compare institutional performance tools for surgical outcome assessment, as the tertiary centres usually receive referrals for moribund patients. Some limitations in developing countries include the lack of uniform methods for comparing data within and between institutes. With the continuous efforts to establish uniform reporting of complications, Clevaino Devine's classification and comprehensive complication index have been introduced and are currently followed at many centres worldwide [29]. Within India, adjusted correction factor usage in patients having a low risk of predicted morbidity (<40%) and mortality (<10%) has been suggested as Jabalpur-POSSUM. The current study supports J-POSSUM as an accurate predictor of morbidities [19]. The author suggested that a J-POSSUM can be used as a validated benchmarking score in specific cases in the Indian setup. However, its generalizability needs to be confirmed by further validation at multiple centres on varied high-risk group patients, highlighting the need for continuous improvement in surgical care.

The strength of this study lies in its comprehensive approach to calculating predictive risk in all surgical categories, including minimal, intermediate, major, and major+ cases. The cases were prospectively studied with sufficient follow-up to validate the equation. However, the study's lacuna is the non-utilization of standard complication categorisation for uniform assessment, and the under-sampling of minor+ and emergency cases restricts generalizability. The low mortality rate in the current study enables risk assessment, which needs further exploration in subsequent analyses.

## Conclusions

An adjusted J-POSSUM, a validated benchmarking score, was used in elective minor, intermediate, and major surgeries. The study revealed a significant decrease in intraoperative and postoperative morbidities (complications) in phase two (p=0.00 and p=0.016), despite having comparable mean OS and PS (p=0.11 and p=0.52). This reduction, a testament to the effectiveness of the interventions, provides reassurance about the quality of care.

It's important to note that due to under-sampling, the P-POSSUM equation for mortality could not be validated in the current study. 
